# Nanobiopolymer for Direct Targeting and Inhibition of EGFR Expression in Triple Negative Breast Cancer

**DOI:** 10.1371/journal.pone.0031070

**Published:** 2012-02-15

**Authors:** Satoshi Inoue, Rameshwar Patil, Jose Portilla-Arias, Hui Ding, Bindu Konda, Andres Espinoza, Dmitriy Mongayt, Janet L. Markman, Adam Elramsisy, H. Westley Phillips, Keith L. Black, Eggehard Holler, Julia Y. Ljubimova

**Affiliations:** 1 Department of Neurosurgery, Cedars-Sinai Medical Center, Los Angeles, California, United States of America; 2 Center for Pharmaceutical Biotechnology and Nanomedicine, Northeastern University, Boston, Massachusetts, United States of America; University of Michigan School of Medicine, United States of America

## Abstract

Treatment options for triple negative breast cancer (TNBC) are generally limited to cytotoxic chemotherapy. Recently, anti-epidermal growth factor receptor (EGFR) therapy has been introduced for TNBC patients. We engineered a novel nanobioconjugate based on a poly(β-L-malic acid) (PMLA) nanoplatform for TNBC treatment. The nanobioconjugate carries anti-tumor nucleosome-specific monoclonal antibody (mAb) 2C5 to target breast cancer cells, anti-mouse transferrin receptor (TfR) antibody for drug delivery through the host endothelial system, and Morpholino antisense oligonucleotide (AON) to inhibit EGFR synthesis. The nanobioconjugates variants were: (1) P (BioPolymer) with AON, 2C5 and anti-TfR for tumor endothelial and cancer cell targeting, and EGFR suppression (P/AON/2C5/TfR), and (2) P with AON and 2C5 (P/AON/2C5). Controls included (3) P with 2C5 but without AON (P/2C5), (4) PBS, and (5) P with PEG and leucine ester (LOEt) for endosomal escape (P/mPEG/LOEt). Drugs were injected intravenously to MDA-MB-468 TNBC bearing mice. Tissue accumulation of injected nanobioconjugates labeled with Alexa Fluor 680 was examined by Xenogen IVIS 200 (live imaging) and confocal microscopy of tissue sections. Levels of EGFR, phosphorylated and total Akt in tumor samples were detected by western blotting.

*In vitro* western blot showed that the leading nanobioconjugate P/AON/2C5/TfR inhibited EGFR synthesis significantly better than naked AON. *In vivo* imaging revealed that 2C5 increased drug-tumor accumulation. Significant tumor growth inhibition was observed in mice treated with the lead nanobioconjugate (1) [P = 0.03 vs. controls; P<0.05 vs. nanobioconjugate variant (2)]. Lead nanobioconjugate (1) also showed stronger inhibition of EGFR expression and Akt phosphorylation than other treatments. Treatment of TNBC with the new nanobioconjugate results in tumor growth arrest by inhibiting EGFR and its downstream signaling intermediate, phosphorylated Akt. The nanobioconjugate represents a new generation of nanodrugs for treatment of TNBC.

## Introduction

Triple negative breast cancer (TNBC) is an aggressive breast cancer phenotype characterized by lack of expression of estrogen receptor (ER) and progesterone receptor (PR), as well as the absence of overexpression of human epidermal growth factor receptor-2 (HER-2) [1].

TNBC often presents as an advanced-stage disease and is treated mostly by systemic administration of conventional chemotherapy due to the lack of specific molecular markers expression [2]. Immunohistochemical analysis showed that TNBC is associated with a high expression of proliferation marker Ki-67 as well as several other markers favoring cancer cell growth, including mutated p53, cyclin E, epidermal growth factor receptor-1 (HER-1, EGFR), vimentin, P-cadherin, and mutated BRCA1.

Anti-EGFR therapy has been increasingly recognized as an important treatment for breast cancer patients [3]. EGFR is a member of the EGFR/ErbB/HER family of type I transmembrane tyrosine kinase receptors including ErbB1/HER-1 (EGFR), ErbB2/HER-2/neu, ErbB3/HER-3, and ErbB4/HER-4 [4], [5]. High expression of EGFR induces erroneous development and unrestricted proliferation in a number of human malignancies, including breast cancer [4]. Tumors overexpressing EGFR represent clinically aggressive cases [6]. They tend to have more rapid cell cycle progression, greater chemoresistance, inhibition of apoptosis, increased angiogenesis, cell motility, and higher rates of metastasis [7]. The clinical data indicated that EGFR expression had a significant prognostic value in TNBC patients [8], with high EGFR levels correlating with poor prognosis. Therefore, EGFR is a potential therapeutic target for the successful treatment of TNBC.

A variety of modalities for blocking EGFR expression and/or function in cancer cells including anti-EGFR monoclonal antibodies (mAbs) and EGFR tyrosine kinase inhibitors (TKI) have been proven effective, particularly when used in combination [4], [5], [7]. However, all of the conventional small molecule drugs are quickly metabolized and cleared through the kidneys, thus requiring high therapeutic concentrations, causing cardio- or other toxicities as side effects. They are also characterized by lack of tumor specificity. Increasingly, nano-based therapeutics have been catching a great deal of attention for cancer treatment. For instance, hyperthermia induced by gold nanoshells sensitized radioresistant TNBC to radiation treatment [9].

The multifunctional polymeric delivery system demonstrated significantly higher antitumor activity in primary and metastatic cancers when compared with drug alone and a pegylated anti-cancer agent [10].

Our aim is to develop an efficient drug delivery system to reach the tumor site specifically, with the ability to carry multiple anti-tumor therapeutic components simultaneously without harmful effects on normal organs. A new nanobioconjugate was designed and synthesized, which specifically delivered anti-EGFR Morpholino antisense oligonucleotides (AON) into breast cancer cells and efficiently inhibited tumor growth *in vivo*. The novel drug is based on poly(β-L-malic acid) (PMLA) platform, which is non-toxic, non-immunogenic, biodegradable, and is a tumor specific drug delivery system with covalently conjugated anti-transferrin receptor (TfR) mAb for transcytosis across the endothelial system [11], [12]. The drugs based on PMLA have been successfully used to treat HER-2 positive breast cancer and gliomas [13], [14], [15], [16]. The nanobioconjugate can target specific cell and receptor types based on the incorporation of mAbs into its structure [17]. PMLA can be covalently conjugated to potential anticancer components such as AONs while allowing them to retain their therapeutic activity [14], [18], [19], [20]. The targeting effect has been enhanced by tandem conjugation of mAbs and anticancer components, making it a novel platform with more efficiently targeted drug delivery [16], [21]. Multiple biomolecules can be attached to the platform allowing the nanobioconjugate to block several molecular markers at the same time to reduce tumor growth, angiogenesis, invasion, and metastasis.

Morpholino AONs that specifically bind to mRNA and inhibit protein synthesis are well established as one of the most powerful specific tools for gene/protein inhibition. Preclinical studies of AONs for cancer treatment showed promising results, and Morpholinos are very stable in plasma making them good candidates for systemic treatment [22], [23], [24]. AONs have been used *in vitro* and *in vivo* and have showed significant inhibition of various genes [24], [25].

The nucleosome-specific mAb 2C5 has been of interest to targeted drug therapy because of its binding specificity toward tumor cells. This mAb is able to recognize and bind to a wide variety of surface bound nucleosomes that are expressed on the tumor cells of multiple cancer cell lines of multiple origins [26], [27]. 2C5 successfully enhanced the distribution of doxorubicin-loaded, long-circulating, polyethylene glycol-coated liposomes (Doxil, ALZA Corp.) in tumors *in vivo* [28].

The present nanobioconjugates were designed to target breast cancer with 2C5 anti-tumor mAb and to inhibit the protein synthesis of EGFR by specific AON. Additionally, a mAb to TfR was attached to allow the nanoplatform to target the tumor vasculature and pass through it. This unique combination of features resulted in a highly specific drug, which accumulated in the tumor tissue and inside tumor cells.

## Results

### 1. Synthesis of Polymalic acid nanobioconjugates

**Figure 1:**
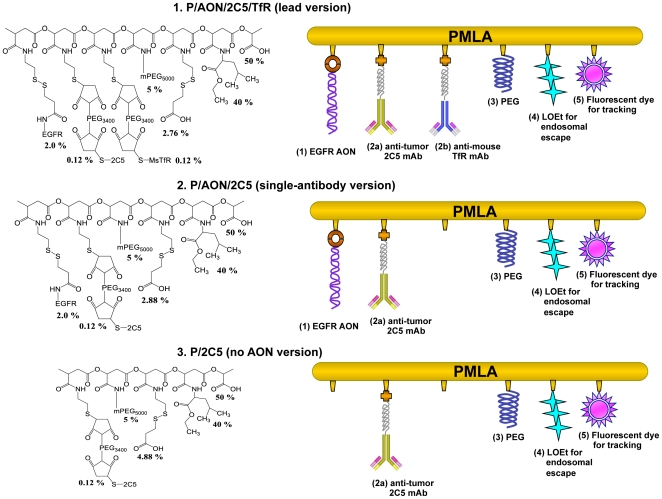
Nanobioconjugate schematic. The new version of nanobioconjugate was designed to inhibit EGFR expression by Morpholino AON *in vitro* and *in vivo*. The components are Morpholino AON to EGFR (1) conjugated to the scaffold by disulfide bonds that are cleaved by glutathione in the cytoplasm to release the free AONs; targeting antibodies 2C5 and TfR either alone or as a combination of mAbs to tumor cells (2a), mouse TfR (2b) for tumor endothelial and cancer cell targeting, and receptor-mediated endocytosis; PEG for drug protection (3); L-leucine ethyl ester together with polymer-COOH (4) for endosomal escape of the drug, and optional fluorescent reporter dye (fluorescein or Alexa Fluor 680) for imaging (5).

The nanobioconjugates were synthesized for TNBC treatment using the hierarchic organization as described previously, with modifications [13]. Of the two sequences of AON specific for EGFR (see materials), version 2 knocked down EGFR expression better than version 1 (data not shown). Thus, we selected version 2 to be conjugated to the polymer platform. The absolute molecular weight of the leading version of nanobioconjugate shown in Fig. 1 was 1,300 kDa determined by light scattering and close to the calculated value based on design. Hydrodynamic diameters (nano sizes) and *ζ* potentials of the nanobioconjugates in Fig. 1 are summarized in Table 1. It has been reported that *ζ* potentials in the range of −4.1 to −5.7 mV should be ideal to allow the nanoparticles to attach to the cell membrane and for nanoparticle internalization [29], [30].

**Table 1:** Nanobioconjugate versions, their sizes, and ζ potentials.

Nanobioconjugates	Size in nm (SD)	Polydispersity index (SD)	Zeta potential in mV
**PMLA**	6.6±0.1	0.1±0.01	−22.9
P/PEG(5%)/LOEt(40%)/MEA(5%)	8.1±0.1	0.79±0.01	−8.45
**Nanobioconjugate 1** P/PEG(5%)/LOEt(40%)/2C5(0.12%)	12.7±0.2	0.69±0.02	−4.13
**Nanobioconjugate 2** P/PEG(5%)/LOEt(40%)/EGFR(2.0%)/2C5(0.12%)	13.0±0.2	0.71±0.02	−3.28
**Nanobioconjugate 3** P/PEG(5%)/LOEt(40%)/EGFR(2.0%)/2C5(0.12%)/MsTfR(0.12%)	14.8±0.2	0.73±0.02	−3.92

### 2. Various tumors express 2C5 antigen and transferrin receptor

**Figure 2:**
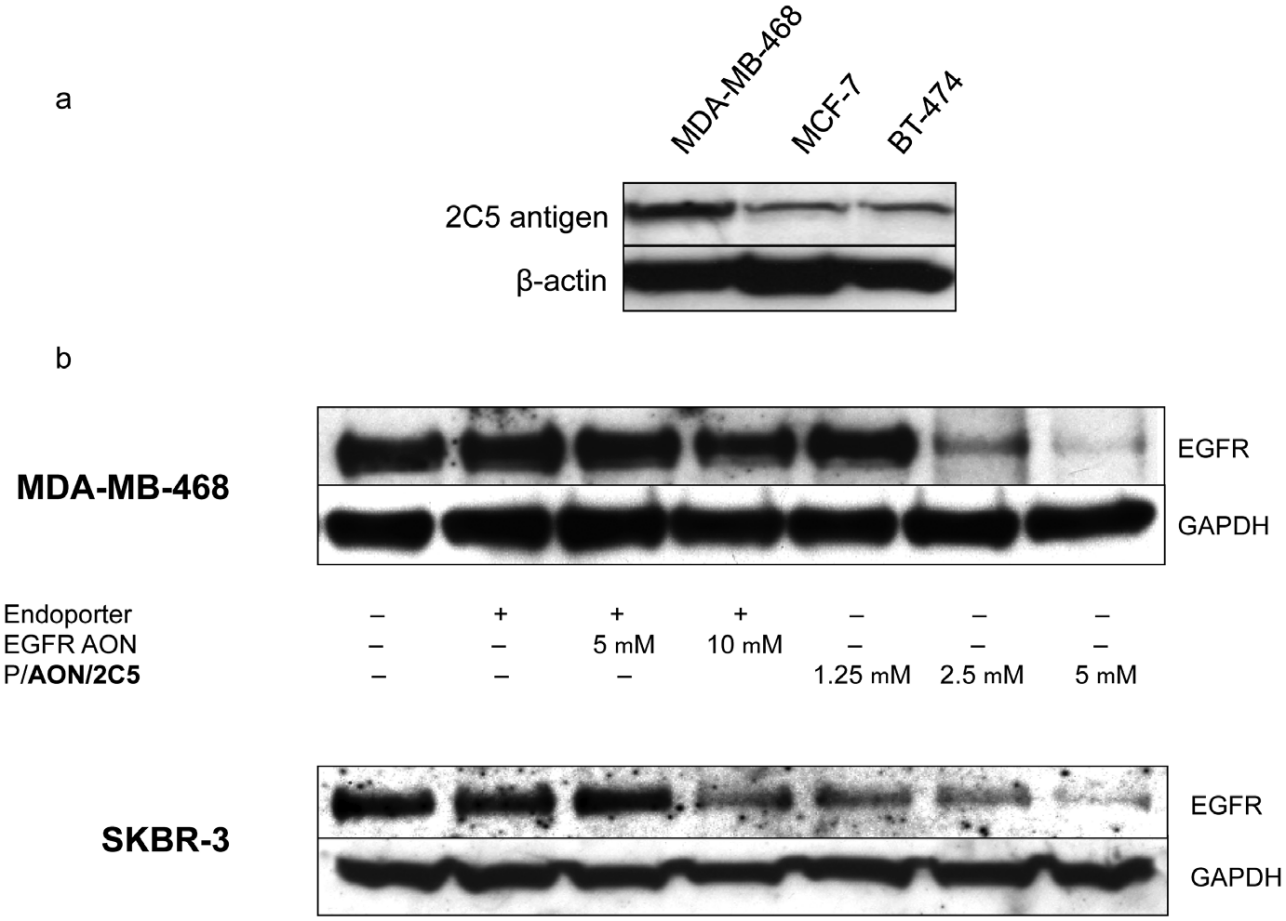
Expression levels of 2C5 and EGFR (after treatment). (2a) Expression level of 2C5 antigen and transferrin receptor in breast cancer cells. 2C5 antigen and TfR expression was studied by western blot analysis. All cell lines expressed TfR and 2C5 antigen. Consistent expression of TfR was seen in all cell lines. 2C5 antigen was also expressed by all cell lines, with the highest expression in MDA-MB-231 (not shown). β-Actin was an internal control to normalize gel loading. (2b) Inhibition of EGFR expression *in vitro* by P/AON/2C5. EGFR overexpressing breast cancer cells MDA-MB-468 (TNBC) and SKBR-3 were treated with either PBS (control), Endoporter (5 µM), two different concentrations of EGFR AON (5 µM or 10 µM) with Endoporter, or three different concentrations of nanobioconjugate (P/AON/2C5) (1.25 µM, 2.5 µM or 5 µM). Two different sequences of EGFR AONs were used for this study (shown in Materials and Methods, 1. Reagents section). Since EGFR AON version 2 inhibited EGFR expression better than version 1 *in vitro*, version 2 (shown here) was chosen for the entire study. 72 hour after treatment, total cell protein was harvested and subjected to Western blot analysis as described in Materials and Methods. Decreased EGFR expression was observed in AON, or P/AON/2C5-treated tumor cells but not in PBS or Endoporter-treated cells. In both cell lines, 10 µM of AON was required to inhibit EGFR. On the other hand, low concentrations (1.25 µM for SKBR-3 and 2.5 µM for MDA-MB-468) of the nanobioconjugate significantly inhibited EGFR expression. GAPDH was an internal control to normalize gel loading.

To investigate the expression level of 2C5 nucleosomal antigen and TfR on different tumors, Western blot analysis was performed to detect 2C5 antigen and TfR on breast cancer cell lines, MDA-MB-468, MCF-7, and BT-474. High TfR expression was seen in all cell lines tested. Although there was some fluctuation in the expression level of 2C5 antigen, it was detected in all cell lines (Fig. 2a). The highest 2C5 antigen expression was seen in MDA-MB-468.

### 3. Nanobioconjugate carrying both tumor targeting 2C5 mAb and EGFR AON (P AON/2C5) significantly inhibits EGFR expression on EGFR positive breast cancer cells in vitro as compared to naked EGFR AON

To evaluate the suppressive effect of nanobioconjugate on the expression of EGFR in high (MDA-MB-468, EGFR+++) and moderate (SKBR-3, EGFR++) EGFR-positive breast cancer cells, western blot analysis was performed. Both EGFR-positive cell lines were treated with PBS (control), AON transduction reagent Endoporter (5 µM), two different concentrations of EGFR AON (5 µM or 10 µM) with Endoporter, or three different concentrations of nanobioconjugate (P /2C5 /AON) (1.25 µM, 2.5 µM or 5 µM by AON). In both MDA-MB-468 and SKBR-3, the EGFR expression was inhibited by high concentration of AON in comparison with the Endoporter control.

P/AON/2C5 also efficiently inhibited EGFR expression in both breast cancer cell lines. Interestingly, low concentrations (1.25 µM for SKBR-3 and 2.5 µM for MDA-MB-468) of nanobioconjugate were sufficient enough to markedly inhibit EGFR expression. Furthermore, 5 µM of nanobioconjugate was more effective than 10 µM of naked AON (Fig. 2b).

### 4. P/2C5 specifically accumulates in the tumor area

**Figure 3:**
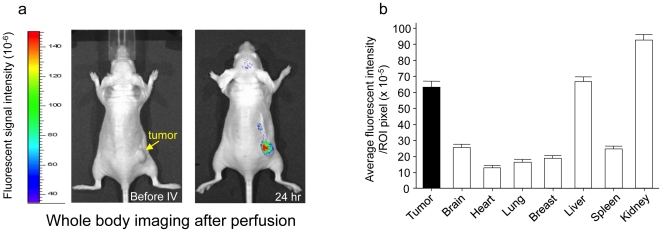
Significant accumulation of P/2C5 in MDA-MB-468 breast tumor *in vivo*. (3a), An MDA-MB-468 subcutaneous tumor-bearing mouse was administered with P/2C5 intravenously. 24 hours later, the animal showed drug distribution mostly in the tumor, as well as in kidney and liver (drug clearing organs). (3b), Other than in kidney and liver, the drug was seen exclusively in the tumor.

The mAb 2C5 has nucleosome-restricted specificity and is able to recognize human tumor cells. 2C5 also works as a specific ligand for liposome targeting to cells *in vitro* and *in vivo* [31]. To confirm its tumor targeting property and successful conjugation of the drug, imaging analysis was performed using the MDA-MB-468 subcutaneous tumor model. The fluorescent signal intensities in the tumor and normal organs were measured and tumor/reference intensity ratios were calculated as described previously [15], [21]. The imaging studies showed that the nanobioconjugate with anti-tumor 2C5 attached to PMLA showed specific drug delivery into implanted human breast tumors. Twenty-four hours after the intravenous injection of drug, it accumulated mostly in the tumor and draining organs, kidney and liver (Fig. 3a). Little drug accumulation was seen in any organs other than the draining organs (Fig. 3b).

### 5. 2C5 antibody shows active tumor targeting effect on MDA-MB-468 breast cancer animal model

**Figure 4:**
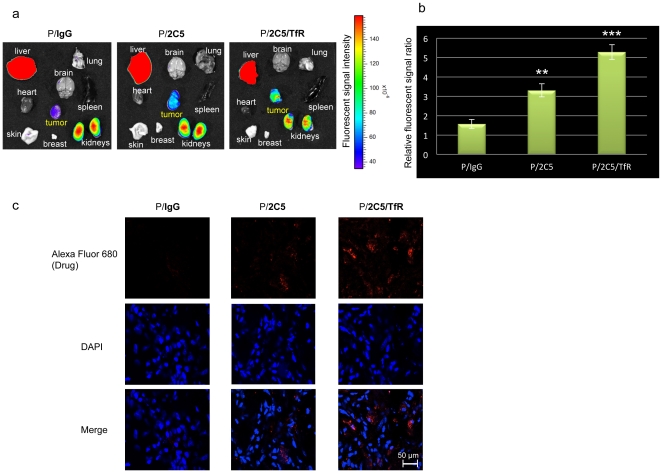
Tumor-specific active targeting effect of the 2C5 antibody *in vivo*. MDA-MB-468 subcutaneous tumor-bearing mice were injected with Alexa Fluor 680-labeled nanobioconjugates (P/IgG, control; P/2C5 or P/2C5/TfR) through their tail vein. The images were taken 24 hours after the drug injection using Xenogen IVIS 200 system. Although the P/IgG barely accumulated in the tumor by EPR effect, the P/2C5 accumulated in the tumor area significantly more than control (Fig. 4a and 4b, **p = 0.001). The highest drug accumulation was seen in P/2C5/TfR treated group where two different tumor targeting antibodies were combined (Fig. 4a and 4b: p = 0.002 vs P/2C5; ***p = 0.0001 vs P/IgG). Confocal microscopy confirmed drug delivery efficiency of the nanopolymer with dual targeting antibodies, P/2C5/TfR (Fig. 4c).

To confirm the tumor specificity of 2C5 mAb covalently attached to the nanoplatform, the Alexa Fluor 680-labeled nanobioconjugates (control P/IgG, P/2C5) or P/2C5/TfR were injected through the tail vein of mice and images were taken 24 hours after the drug injection. Control polymer with IgG showed only weak signal in the tumor (Fig. 4a). In contrast, P/2C5 showed higher accumulation in the tumor area in comparison with P/IgG. Finally, P/2C5/TfR showed significantly enhanced accumulation in the tumor in comparison to the other two groups as determined in three independent experiments (Fig. 4a). Subsequent confocal microscopy was performed on sections of tumors removed 24 hours after intravenous injection of Alexa Fluor 680-labeled drugs. Again, higher drug accumulation was observed inside cancer cells in the case of P/2C5 as compared with control. The highest drug accumulation was seen in the P/2C5/TfR injected group (Fig. 4b).

### 6. The lead drug significantly suppresses EGFR-positive triple negative breast tumor growth in vivo

We investigated the therapeutic effect of the nanobioconjugate upon intravenous treatment using subcutaneous mouse models of human breast tumor xenografts. We used the MDA-MB-468 cell line that overexpresses EGFR for *in vivo* treatment experiments. MDA-MB-468 tumor-bearing mice were treated with either PBS (control), P (polymer alone, control), P/2C5, P/AON/2C5, or P/AON/2C5/TfR. No body weight loss, morbidity, or death was observed, indicating that all treatments were well tolerated (data not shown).

**Figure 5:**
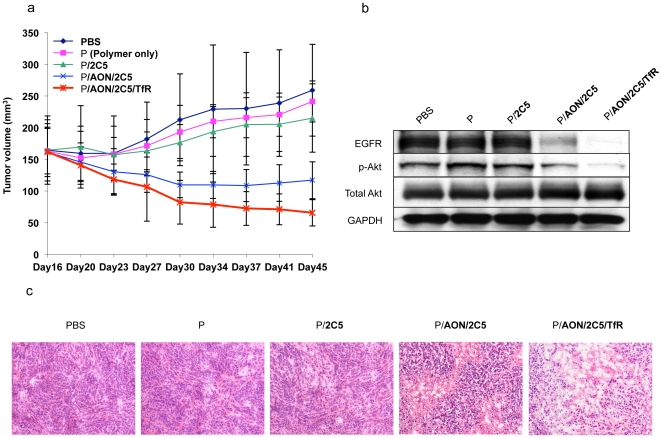
Tumor inhibition in mouse model, and effects on EGFR expression and Akt phosphorylation. (5a) Tumor growth inhibition in mice. MDA-MB-468 subcutaneous breast tumors treated systemically with P/AON/2C5, or P/AON/2C5/TfR were significantly inhibited compared with PBS (control), P(polymer only), or P/2C5 (without anti-tumor component) (P<0.03). The highest inhibition of tumor growth was observed in mice treated with P/AON/2C5/TfR (p<0.03 vs. controls; p<0.05 vs. P/AON/2C5). Error bars denote SEM. (5b) Expression of EGFR and phosphorylated Akt (p-Akt) after treatment of EGFR-positive tumors *in vivo*. Western blot analysis showed the decrease in EGFR and p-Akt expression in P/AON/2C5-, or P/AON/2C5/TfR -treated mice compared to controls. The highest inhibition of EGFR and p-Akt was seen upon treatment with P/AON/2C5/TfR where 2 targeting antibodies were combined. GAPDH was an internal control to normalize gel loading. (5c) Histopathological analysis of tumors. Hematoxylin and eosin staining of tumors treated with PBS, P, P/2C5, P/AON/2C5 or the leading drug P/AON/2C5/TfR. Consistent with tumor size reduction data, the leading drug P/AON/2C5/TfR treated tumor showed significant reduction in the number of viable tumor cells as compared to other treatments.

There was no anti-tumor effect in the PBS-, P-, or P/2C5-treated mice. Mice treated with P/AON/2C5 showed significant breast tumor inhibition compared to the above-mentioned groups. The most profound anti-tumor effect was observed when mice were treated with the lead drug with two targeting antibodies, P/AON/2C5/TfR (Fig. 5a). The leading drug showed the highest degree of inhibition of tumor growth within all treatment groups with an enhanced tumor inhibitory effect compared to single-antibody treatments (Fig. 5a; P<0.05 vs. P/AON/2C5; P<0.03 vs. all other treatment groups). Moreover, the tumor growth inhibition seen in mice treated with either P/AON/2C5 or P/AON/2C5/TfR within the first four days after the initial treatment is noteworthy.

Hematoxylin and eosin staining (Fig. 5c) showed decreased numbers of viable cells in P/AON/2C5 treated tumors. Furthermore, there was significant necrotic change in the case of P/AON/2C5/TfR treated tumors.

Western blot analysis using lysates of subcutaneous MDA-MB-468 breast tumors after different treatments was performed to evaluate whether the nanobioconjugate with AON inhibits EGFR expression and its related signal. Tumor EGFR expression was indeed inhibited by P/AON/2C5 (Fig. 5b). P/AON/2C5/TfR, where two mAbs were combined, caused the highest inhibition of EGFR expression in the tumor. Phosphorylated Akt, which is one of the main downstream molecules in the EGFR-regulated signaling pathway, was also reduced by the nanobioconjugates. The version of nanobioconjugate with two antibodies (the lead drug) showed the most dramatic decrease in the expression of p-Akt signal (Fig. 5b), which supports the idea that the lead drug inhibited breast cancer growth by blocking the EGFR-regulated pathway. Total Akt expression was not changed by any treatment.

## Discussion

We have previously shown that our nanobioconjugate drug delivery system with PMLA as a platform specifically targeted cancer cells and inhibited brain tumor growth and angiogenesis [32], [33]. A specific nanobioconjugate variant also significantly inhibited HER-2 positive breast cancer growth both *in vitro* and *in vivo* [16]. In this study, we investigated the therapeutic effects of the nanobioconjugate carrying a combination of EGFR AON and 2C5 anti-tumor mAb along with TfR as active targeting for EGFR-positive triple negative breast cancer treatment.

Breast cancer is the most common malignancy of women in the U.S. and is the second leading cause of cancer mortality [34], [35]. Unlike patients with ER/PR-positive or HER-2-overexpressing breast cancer, systemic treatment options for patients with TNBC are limited due to the lack of a molecular target [1]. Recently, a number of targeted agents have been considered for TNBC including poly(ADP-ribose) polymerase (PARP) inhibitor, mTOR (mammalian target of rapamycin) inhibitor, anti-VEGF mAb, and anti-EGFR mAb [3].

EGFR activates downstream signaling pathways, such as the phosphoinositide-3 kinase (PI3K)/Akt pathway, which mediates cell proliferation, survival, and migration [36], [37]. Anti-EGFR therapy has been increasingly recognized as a potential treatment for breast cancer patients, and recently, advances in this direction have been made [5], [38]. Furthermore, EGFR is an independent prognostic factor for patients with TNBC [8]. Therefore, EGFR-targeted therapy is one of the promising strategies for TNBC treatment and can potentially improve therapeutic outcome in TNBC patients.

Several novel EGFR inhibitors are currently in pre-clinical and clinical development including mAbs and small-molecule tyrosine kinase inhibitors. Gefitinib (Iressa™, AstraZeneca) is a small-molecule tyrosine kinase inhibitor of EGFR that binds reversibly to the ATP-binding site of EGFR. However, phase II trials of Gefitinib in refractory metastatic breast cancer yielded disappointing responses [39]. Cetuximab (Erbitux™, ImClone Systems Incorporated) is a mAb that binds with high affinity to the extracellular domain of EGFR, competes for ligand binding and blocks activation of the receptor by EGF or TGF-α. It also induces antibody-mediated receptor dimerization resulting in receptor downregulation [38], [40]. However, the phase I trial of Cetuximab in combination with Paclitaxel against advanced breast cancer was not considered promising [41]. Recently, the dual kinase inhibitor, Lapatinib, which possesses tyrosine kinase receptor inhibitory activity against both EGFR and HER-2, appeared to improve the current model of tyrosine kinase inhibition and became a novel anti-EGFR therapy [42]. However, despite this progress in anti-EGFR based cancer treatments, they lack of tumor-specific delivery, which should result in inefficient anti-tumor activity. For therapeutic effect, high doses are needed, which lead to serious toxicity. The nanobioconjugates are ideal drug delivery tools with enhanced specificity to the tumor and are capable of carrying multiple drug combinations on a single platform. The drug delivery efficiency is significantly improved when the nanopolymer is combined with multiple components such as targeting antibodies against a tumor marker by exploiting both passive targeting through EPR effect and active targeting by mAbs [43], [44], [45].

The synthesis of the complete nanobioconjugate on polymalic acid nanoplatform with covalently attached EGFR AON in combination with 2C5 and TfR mAbs has been successfully achieved (Fig. 1). AON is a stable and promising option for cancer treatment [24], [25]. AON blocked EGFR on glioma cells as good as siRNA resulting in significant tumor growth inhibition *in vivo* and *in vitro* [46]. As Table 1 shows, the size (smaller than 30 nm) of these conjugates and their slightly negative *ζ* potential make them ideal for the interaction with the cell membrane and intracellular internalization. In addition, the homogeneities of each product were reflected by their polydispersity index.

Western blot analysis showed that P/AON/2C5 significantly inhibited the expression of EGFR *in vitro* (Fig. 2b). The low dose of nanobioconjugate was more effective than the high dose of naked AON. This result suggests that the nanobioconjugate is a very efficient delivery vehicle in terms of drug uptake and efficacy. More importantly, unlike commercially available transduction agents, PMLA is non-toxic and non-immunogenic, which is ideal for systemic treatment.

It has been shown that 2C5 specifically targets tumor cells, and thus it increases anti-cancer effect and reduces side effects of the treatment [27], [28], [31]. In accordance with previously published data, the nanobioconjugate carrying 2C5 mAb specifically recognized the tumor, which suggests that the activity of the tumor targeting 2C5 antibody was not changed during chemical synthesis. A new version of nanobioconjugate carrying tumor targeting 2C5 mAb and EGFR AON, P/AON/2C5, significantly inhibited breast tumor growth *in vivo* (Fig. 5). Breast tumor growth was inhibited even more when the animals were treated with P/AON/2C5/TfR where two targeting antibodies were combined on one nanobiopolymer. Previously, we have proven that tandem configuration of specific antibodies enhanced tumor targeting [16], [21]. Therefore, targeting tumor vascular endothelium by mouse anti-TfR mAb in combination with anti-tumor 2C5 mAb significantly enhanced tumor targeting effect of the nanobioconjugate.

Western blot analysis using tumor samples proved that the nanobioconjugate efficiently delivered the AONs via attached targeting antibodies into the tumor cells and this explained the significant treatment effect observed with novel nanobioconjugate to inhibit the growth of TNBC. A plausible mechanism of action of this nanobioconjugate appears to be the inhibition through EGFR block of Akt phosphorylation, which is a downstream target of EGFR. A similar blockage of Akt phosphorylation in breast cancer was also observed after successful treatment of HER-2-positive breast cancer-bearing mice using another nanobioconjugate that inhibits HER-2 synthesis and activity [12].

The results of this study suggest that tumor-targeted nanobioconjugate carrying EGFR AON with significant anti-tumor activity against EGFR-positive TNBC may represent a new generation of cancer therapeutics with a potential for efficacy against triple negative breast cancers.

## Materials and Methods

### 1. Reagents

Two versions of Morpholino™-3′-NH2 antisense oligonucleotides to EGFR were custom made by Gene Tools (Philomath, OR):

Version 1: 5′- GGTCGCATCGCTGCTCCCCGAAGAG-3′,

Version 2: 5′- TCGCTCCGGCTCTCCCGATCAATAC-3′

Highly purified, endotoxin-free poly(β-L-malic acid), Mw (weight-averaged) = 100 kDa, polydispersity = 1.1, was obtained from the culture broth of *Physarum polycephalum*. Rat anti-mouse TfR mAb R17217 (mTfR) was purchased from Southern Biotech (Birmingham, AL). Cysteamine (2-mercaptoethyl-1-amine hydrochloride), N-hydroxysuccinimide, other reagents and solvents were of highest available purity and purchased from Sigma-Aldrich (St. Louis, MO).

Mouse autoimmune mAb 2C5 recognizing tumor cell surface-bound nucleosomes released from neighboring apoptotic tumor cells was a gift from Prof. V.P. Torchilin (Northeastern University, Boston, MA).

### 2. Synthesis of polymalic acid nanobioconjugates

The nanobioconjugates contain five to six key components (Fig. 1): PMLA as the backbone of delivery carrier, Morpholino AON for the inhibition of EGFR protein synthesis, tumor vasculature targeting anti-mouse TfR mAb, targeting anti-tumor 2C5 mAb, 40% leucine ethyl ester as an endosome escape unit to achieve cytoplasmic AON delivery, and 5% PEG_5000_ to increase stability in the bloodstream. The preconjugate containing 40% leucine ethyl ester, 5% PEG_5000_ and 10% of cysteamine (% referring to the total amount of carboxyl groups in PMLA) was synthesized by the methods described previously [13]. The antibodies attached to the preconjugate were qualitatively and quantitatively assayed by size exclusion HPLC. ELISA with purified TfR and 2C5 antigen was used to verify functional reactivity of attached antibodies as described. Conjugates for imaging were fluorescently labeled with Alexa Fluor® 680 C2-maleimide (Invitrogen, Carlsbad, CA) by forming thioether with sulfhydryl groups. Conjugate without AONs and mAb was used as a control.

### 3. The nanobioconjugate characterization

Chemical and physical characterization of nanobioconjugate was performed by various methods including L-malate dehydrogenase assay, PEG colorimetric determination and protein quantification, size and *ζ* potential, HPLC, and ELISA. HPLC was performed on a Hitachi analytical Elite LaChrom HPLC-UV system and size exclusion column BioSep-SEC-S 3000 column. The nanobioconjugate variants were characterized with respect to their size (hydrodynamic diameter) on the basis of noninvasive back-scattering (NIBS) and ζ potential from electrophoretic mobility based on the Helmholtz-Smoluchowski formula, using electrophoresis M3-PALS [19]. Both measurements were performed in a Zetasizer Nano System ZS90 (Malvern Instruments, Malvern, UK). Data on molecular size and *ζ* potential from three independent measurements represent mean ± standard deviation.

### 4. Cell lines and culture conditions

Human breast cancer cell lines MDA-MB-468 (TNBC, EGFR positive), SKBR-3 (EGFR-positive), BT-474 (EGFR-positive), and MCF-7 (EGFR-negative) were obtained from American Type Culture Collection (Manassas, VA). SKBR-3 was cultured in McCoy's 5A medium with 10% fetal bovine serum and antibiotics. All other cell lines were cultured in DMEM with the same supplements.

### 5. Nomenclature

The term “nanobioconjugate” denotes the drug delivery system with PMLA as the nanoplatform and various functional groups covalently attached to it, specifically the rat anti-mouse TfR mAbs, and leucine ethylester (LOEt) as the endosomal escape unit. The newly synthesized versions of nanobioconjugate to treat EGFR-positive breast cancer contained a single anti-cancer drug (EGFR AON) with either one mAb (2C5) or two mAbs (2C5 and TfR). All versions of nanobioconjugates used in this study are shown in Fig. 1 and Table 1.

### 6. Western blotting

The protein samples were harvested from a variety of breast cancer cells (shown in Fig. 2.) and the expression level of 2C5 antigen and TfR were detected by western blotting as described previously [47]. MDA-MB-468 EGFR-positive TNBC cells and EGFR-positive SKBR-3 breast cancer cells were treated with one of the following: PBS (control), Endoporter (control, transduction reagent), AON to EGFR with Endoporter, and P/AON/2C5. Cell lysates were collected 72 hours after treatment. EGFR and glyceraldehyde 3-phosphate dehydrogenase (GAPDH, to normalize gel load) expression was analyzed by western blotting.

MDA-MB-468 TNBC subcutaneous breast tumor bearing mice were treated with controls (PBS); P(Polymer control), P/2C5, P/AON/2C5, and P/AON/2C5/TfR. Cell lysates were prepared from excised breast tumor samples and analyzed by western blotting to detect EGFR, total Akt, phosphorylated Akt (p-Akt), and GAPDH (for equal gel loading). All of the antibodies used were purchased from Cell Signaling Technology (Beverly, MA).

### 7. Tumor xenografts in nude mice

All experiments with animals were performed in accordance with the protocols approved by the Cedars-Sinai Medical Center Institutional Animal Care and Use Committee (IACUC). Athymic mice were purchased from Taconic (Hudson, NY). A total of 1×10^7^ MDA-MB-468 cells suspended in 150 µl of Matrigel (BD Biosciences, Bedford, MA) were injected into the right flanks of 35 mice (5 mice per group), and treatment was commenced when tumors reached an average size of >120 mm^3^ (21 days after injection). Mice were divided into 5 treatment groups and administered either sterile PBS (control), P (Polymer control), P/2C5, P/AON/2C5, or P/AON/2C5/TfR through the tail vein twice a week. Treatments were performed 8 times (for 4 weeks).

Tumor sizes were measured with calipers twice a week, and tumor volumes were determined using the formula: (length×width^2^)×(π/6).

Four days after the last treatment, the animals were anesthetized with 3% isoflurane-air mixture and sacrificed by cervical dislocation. Tumor samples were stained with hematoxylin and eosin for morphological observation.

The same subcutaneous tumor model was used for the imaging study.

### 8. In vivo imaging study

To assess the organ localization of nanobioconjugates, 1×10^7^ MDA-MB-468 human breast cancer cells suspended in 150 µl of Matrigel were implanted into the right thigh of athymic mice (CrTac:NCr-Foxn1nu Homozygous, Taconic) as described above.

When tumors grew up to 120 mm^3^, 160 µl of Alexa Fluor 680-labeled Polycefin variants were injected intravenously at the concentration of 4 µM. PMLA with IgG, P/IgG, was used as a control. For the assessment of drug distribution and localization in nude mice, animals were studied in a Xenogen IVIS 200 imager under isoflurane anesthesia at different time points (before drug administration, 1 h, 3 h, 6 h, and 24 h after the injection of the drug). Twenty-four hours after drug administration, mice were euthanized. Intra-arterial PBS perfusion was done in order to wash out the circulating drugs in blood vessels. The tumor and major organs were harvested to detect the fluorescent signal. The fluorescent signal intensities in the tumor and different organs were analyzed by Xenogen Living Image® software, Version 2.50 (WaveMetrix, USA).

### 9. Confocal microscopy

To assess the tissue localization of nanobioconjugates, tumor-bearing mice were injected through the tail vein with either Alexa Fluor 680-labeled P/IgG (control), Alexa Fluor 680-labeled P/2C5, or Alexa Fluor 680-labeled P/2C5/TfR, as above. Twenty-four hours after drug administration, mice were euthanized, tumors were harvested, snap-frozen in liquid nitrogen, and embedded in OCT compound for the fluorescent signal detection on sections by confocal microscopy (TCS SP5 X microscope; Leica Microsystems, Mannheim, Germany).

### 10. Statistical analysis

Student's t-test (for two groups) and analysis of variance (ANOVA, for three or more groups) were used to calculate statistical significance of the experimental results. GraphPad Prism4 program (GraphPad Software, San Diego, CA) was utilized for all calculations. Data are presented as mean ± standard error of mean (SEM). The significance level was set at P<0.05.
